# Cerebral Surgery

**Published:** 1905-10-28

**Authors:** 


### CEREBRAL SURGERY.

**Operations for the Exploration of the Contents of the Spinal Canal.**—As in cerebral surgery, many surgeons have discarded the trephine and only use the osteoplastic method of opening the skull, so in more recent times it has been suggested that a similar change ought to take place in our methods of exposing the spinal cord. The ordinary way of accomplishing this is by removing a varying number of vertebral spines and laminæ after having bared them through a median incision. But if certain preliminary details be observed it is equally easy to turn up the spines and laminæ embedded in the overlying soft tissues, thus giving free exposure of the cord and subsequently to replace the bony parts, so that they re-unite to form an intact vertebral column. The advantages of this are obvious, insuring, as it does, the unimpaired strength of the vertebral column and shutting off the spinal cord more completely from a chance of infection through the wound. The patient is placed in the semi-prone position and the spines of the vertebræ which are to be removed carefully located. As a preliminary to the turning up of an osteoplastic flap, the spine of the vertebra above those to be contained in the flap is removed. This is done through a vertical incision, and, after severing its ligamentous connections with scissors, the spine is sawn off as close to its base as possible by a Gigli's saw. The object of this is to prevent the locking of the spines in the flap with those above when the former is turned up. Two parallel incisions are then made on either side of the mid-line and midway between the tips of the spinous and transverse processes. These incisions curve inwards to meet each other below the lowest spine which is to be turned up. The incision is deepened through aponeurosis and muscle down to the bone, which is cleared of periosteum by a wide chisel. The bones are then cut through by parallel saw cuts. Doyen's saw, which is practically a modified Hey's saw provided with a guard that limits the depth of the incision, is the best instrument to use. The ligaments connecting the lowest vertebræ in the flap with that below it being severed, the whole flap can be turned up and a full exposure of the cord in its membranes made. After the examination of the cord is completed the whole flap is replaced and the muscular layers accurately apposed by deep-buried catgut sutures and the skin by silkworm gut. But as an objection to this method, which is described and illustrated in great detail by Bickham,[1] it may be pointed out that it is necessary to know beforehand exactly how many laminæ are to be removed, and this knowledge is absent in so many operations on the cord which are of an exploratory nature. Further, the importance of the spines and laminæ for the maintenance of the integrity and strength of the vertebral column is probably much over-estimated. Considering that the bodies of the vertebræ, their transverse and articular processes are untouched, the mere removal of the laminæ can cause but little weakness in the spine. And, lastly, the great argument in favour of osteoplastic operations on the cranium—namely, the protection of the nerve-centres from injury subsequent to the operation, does not obtain in the case of the cord, because the latter is so deeply placed. But in spite of these objections the method will probably be widely adopted in the future in those cases where it is known beforehand that a large area of cord has to be exposed. And especially will this be the case in the lower regions of the spine where the vertebræ have to bear so large a proportion of the bodyweight and where the integrity of the spinous processes is important because of important muscular origins and attachments. Such a case, for example, is described by Williamson.[2] A man was shot and the bullet lodged in the lumbo-sacral region of the spine. No motor paralysis resulted, but the tendo achillis reflexes were absent and there was partial tactile anæsthesia in the perineum. There was incontinence of urine and retention of rectal contents. Very severe sacral pain formed the most prominent subjective symptom. The bullet was removed after being localised by the Röntgen rays. The pain ceased, the functions of the bladder and rectum returned, and the sensory disturbance round the anus recovered partially.

[1] Annals of Surgery, March 1905. [2] Med. Chron., April 1905.

